# Taping for jump performance: The effects of various knee taping techniques on a drop jump

**DOI:** 10.17159/2078-516X/2025/v37i1a20724

**Published:** 2025-09-15

**Authors:** K Szeles, A Green

**Affiliations:** Department of Sport and Movement Studies, Faculty of Health Science, University of Johannesburg, South Africa

**Keywords:** biomechanics, knee strapping, reactive strength, kinematics

## Abstract

**Background:**

Drop jump landing (DJL) analysis is used to identify an athlete’s reactive strength abilities and landing mechanics. The aim of the study was to investigate the effects of different knee taping conditions on lower limb kinetics and kinematics during a DJL.

**Objectives:**

The aim of the study was to investigate the effects of different knee taping conditions on lower limb kinetics and kinematics during a DJL.

**Methods:**

Twenty-one athletes (age: 22.0±2.7 years; height: 1.68±0.08 m; mass: 63.3±10.6 kg) performed a series of 40cm drop jumps under four taping conditions – no tape (NT), dynamic tape (DT), rigid tape (RT), and kinesio-tape (KT). Reactive strength indices (ground contact time (GCT), Landing impact force, and reactive strength index (RSI)) were compared across conditions to determine reactive strength performance. Continuous biomechanical data were analysed using SPM1d repeated measures ANOVA (p<0.05).

**Results:**

Significant changes were observed in ground reaction force (GRF) (3–7%, p=0.001) between NT vs RT and KT, GCT (p=0.003) between NT vs KT, RSI (p=0.013) between NT vs KT, peak ankle abduction (p=0.05) and peak knee rotation (p<0.001). SPM1d analyses revealed significant changes in ankle rotation (1–6%; 17–20%; 67–100%), knee abduction (14–16%; 49–51%) and rotation (1–15%; 17–40%; 51–100%), and hip rotation (16%; 71–100%).

**Conclusion:**

The knee taping conditions showed minor kinematic changes to the ankle, knee, and hip joint angles. Further, the kinetics revealed that KT showed the most improvement in reactive strength performance, having the highest GRF, contributing to SCC efficiency, and the shortest GCT, yielding the highest RSI. This suggests that KT on the knee may contribute to reactive strength performance in a DJL.

The drop-jump landing (DJL) test is a screening tool used to measure the overall lower limb alignment in the coronal plane to detect abnormal (valgus) positions, forces on landing, and reactive strength.^[1]^ DJLs mimic the rapid deceleration followed by a maximal vertical jump observed in rebounding tasks,^[1]^ such as in a game of netball. A netball player’s ability to repeatedly tolerate moderately high loads upon landing throughout a match is crucial and largely depends on the concentric and eccentric strength of the lower limb muscles.^[2]^ Previous research shows that higher eccentric strength leads to better biomechanical efficiency during a DJL, as seen by improved absorption and reuse of external loads, leading to optimised GRFs and joint moments.^[3]^

The Reactive strength index (RSI), ground contact time (GCT), and ground reaction forces (GRF) are the performance metrics used when assessing a DJL, where higher RSI values correlate with better jump performance and are characterised by increased jump height and shorter GCT.^[4,5]^ Furthermore, RSI can be used to reflect an athlete’s ability to efficiently transfer force during a jump.^[6]^

The DJL profile consists of two main phases: the drop jump (DJ) and the drop landing (DL), which allows analysis of landings in each phase.^[1]^ These phases can be broken down into six sub-phases: eccentric, concentric, flight, loading, attenuation, and control.^[4]^ The DJ phase includes eccentric, concentric, and flight sub-phases. The eccentric phase involves the descent of the centre of mass (COM) from the drop until velocity reaches zero.^[4]^ The concentric phase covers the ascent of COM from zero velocity to take-off, leading into the flight phase, which lasts until the second landing.^[4]^ The DL phase comprises the loading, attenuation, and control sub-phases. The loading phase spans from initial ground contact to the peak vertical ground reaction force (VGRF).^[4]^ The attenuation phase follows, lasting from the peak VGRF to the local minimum VGRF.^[4]^ Lastly, the control phase extends from the local minimum VGRF until vertical COM velocity crosses zero.^[4]^

Traditional biomechanical analysis has often focused on discrete points, such as peak or minimum kinematic and kinetic values, overlooking the dynamic nature of the full movement cycle as joints progress through their full range of motion and forces develop and attenuate.^[7]^ One-dimensional Statistical Parametric Mapping (SPM1d) has become increasingly used for biomechanical data on tasks such as landing, for example a study on the effects of kinesio taping on DJL by Limroongreungrat and Boonkerd^[8]^, as it offers a more comprehensive approach by analysing entire kinematic and kinetic sequences, enabling the detection of subtle movement nuances and performance traits that may be missed with traditional discrete methods.^[7]^ Unlike discrete analysis, SPM1d can identify differences across the entire movement trajectory rather than at isolated time points. These differences are typically reported as statistically significant clusters within the time-normalised movement cycle, highlighting specific phases or percentages of the movement cycle where deviations occur.

Athletes use various taping methods to enhance performance and improve force attenuation, each with distinct mechanisms of action. The most common types are rigid tape (RT), kinesio tape (KT), and the more recently developed dynamic tape (DT). RT stabilises joints and muscles by restricting movement, limiting excessive range of motion, and protecting injured areas.^[9]^ Conversely, KT mimics skin elasticity to allow natural movement while promoting circulation, reducing pain, enhancing proprioception, and supporting muscles and joints.^[8]^ Lastly, DT, similar to KT but with greater elasticity and multidirectional stretch, offers stronger recoil for improved control and load accommodation on tissues.^[9]^

Previous research highlights the potential effects of different taping types on joint angles, GRF, power, and proprioception. KT and DT applied to the knee during a DJL significantly reduced dynamic knee valgus (DKV), while RT decreased GRF and shear forces.^[10,11]^ Reductions in DKV and GRF are associated with a lower risk of soft tissue knee injuries.^[12]^ Taping has also enhanced jump performance, with KT over the knee and quadriceps significantly increasing vertical jump height and power output.^[13]^ Additionally, KT improved proprioception in individuals with poor proprioception during Biodex balance tests.^[14]^ Despite the reported benefits of RT, DT, and KT, no research has directly compared the effects of these tape types on performance.

The study aimed to investigate the effects of different taping conditions on knee joint angles and reactive strength during a DJL.

## Methods

### Participants

The calculated sample size was based on a large effect size (CI 95%; *f*=0.4; η^2^≈0.5; and a power of 0.95), resulting in a minimum sample size of 15 participants. Twenty-one injury-free female netball players (age: 22.0±2.7 years; height: 1.68±0.08 m; mass: 63.3±10.6 kg) participated in this study. Participants signed an informed consent form, and institutional ethics approval was granted prior to the commencement of the study (REC-1760-2022).

### Procedure

Participants were required to perform three drop jump landings (DJL) off a 40cm box under four knee-taping conditions: No Tape (NT), Rigid Tape (RT), Dynamic Tape (DT), and Kinesio Tape (KT). Participants were instructed to place their hands on their hips, step off a 40 cm box, land with feet in a toe-first landing pattern on separate force plates, followed immediately by a counter-movement jump (CMJ) by jumping vertically upwards as high and quickly as possible before landing again on the force plates.^[15]^

### Biomechanical analysis

All kinematics were captured using the model CGM 2.5 with a 10-camera system recording at 200Hz (Vicon Vero 2.2 (Vicon Motion Systems Ltd, UK)). The calibration and 3D tracking was done using Nexus software (Vicon Motion Systems Ltd, UK). Multi-axis force plates (Bertec, Columbus, Ohio, USA) were used to measure the ground reaction forces at 1000 Hz.

### Taping allocation

Three tapes were assessed: The RT and DT conditions used Mulligan’s Tape technique and KT used the “Y” formation technique. The same practitioner applied all taping to ensure consistency. Jump protocols were performed within 5 minutes of tape application.

**Fig. 1:**
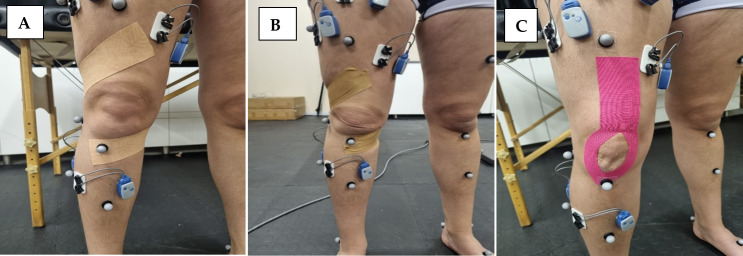
A: Mulligans tape technique using dynamic tape. B: Mulligans tape technique using rigid tape. C; The “Y” formation technique using kinesio tape.

In the Mulligans Tape technique (Figure 1A & B) the individual was placed with the knee flexed at a 25° angle and the hip in a slight internal rotation, where tape was applied from the fibula head, passing over the tibia while applying an internal tibia torsion force.^[11]^ The tape then passed posterior, inferior to the medial knee joint line, and centrally over the posterior aspect.^[11]^

In the “Y” formation technique (Figure 1C), the individual was prone with their hip in a neutral position and the knee flexed with the quadriceps in a maximum stretch position. ^[14]^ The tape was cut in a “Y” formation and applied from the anterior inferior iliac spine to the tibial anterior tuberosity with the tension placed between 50 and 75% in the middle without any stretch in the extremities.^[14]^

### Data analysis

The jump profile (DJL initiation to termination) was divided into six phases: eccentric (0–4%), concentric (4–23%), flight (23–25%), loading (25–42%), attenuation (42–70%), and control (70–100%).^[4,7]^ For each DJL trial, subjects descended from the platform, landed with each foot on a separate force plate (initial landing), immediately performed a maximal vertical jump, and then landed again (second landing).^[6]^ Kinetics and kinematics of the three landing attempts were time-corrected and averaged. Jump Height was calculated using the momentum-impulse methods, second integral of net acceleration. Ground contact time (GCT) was calculated as the duration from the initial ground contact to the point of take-off.^[7]^ The Reactive strength index (RSI) was calculated as jump height divided by jump time.^[7]^ Custom-written algorithms were used to analyse body positions as derived from raw marker location data in MatLab (Mathworks, Natick, Massachusetts, USA).

### Statistical analysis

Discrete DJL metrics: RSI, GCT, jump height, and max take-off velocity were evaluated for normality using Shapiro-Wilk test and subsequently compared using Friedman’s tests with multiple comparison post-hoc test in SPSS (p<0.05) where significance levels were determined (*W* = 0.1 – <0.3 (small effect), 0.3 – <0.5 (moderate effect) and >=0.5 (large effect)) (*n^2^* = 0.1 – <0.3 (small effect), 0.3 – <0.5 (moderate effect) and > = 0.5 (large effect)). Peak joint angles were normalised to the NT condition and compared using Friedman’s tests. Continuous biomechanical data were analysed using nonparametric SPM1d repeated measures ANOVA (p<0.05) in MatLab.^[7]^ Post hoc analyses of continuous data were conducted in the form of non-parametric paired t-tests using a Bonferroni correction p value=0.0085.

## Results

DJL variables are reported in Table 1. No significant differences were noted in the jump height (p=0.921, η^2^=0.008). However, small differences were noted in ground contact time (p=0.003, η^2^ =0.025) (Table 1) between NT and KT (p=0.001), as well as in RSI (p=0.013, *W*=0.225) between NT and KT (p=0.006). Maximum and relative landing impact force (p=0.21, *W*=0.08) were not significantly different across the taped conditions and all resulted in a small effect size (Table 1).

The normalised peak joint angles (Table 2) revealed small differences in ankle abduction (p=0.05, η^2^=0.158) between DT vs KT (p=0.045), with no differences found in ankle flexion (p=0.20, η^2^=0.086) and ankle rotation (p=0.54, η2=0.025). Small differences were also found in knee rotation (p<0.001, *W*=0.532) between RT vs KT (p<0.001) and DT vs KT (p<0.001). No differences were seen in knee flexion (p=0.73, η^2^=0.013) or knee abduction (p=0.62, *W*=0.025), and no significant differences were found in hip flexion (p=0.07, *W*=0.144), hip abduction (p=0.48, η^2^=0.027) and hip rotation (p=0.33, *W*=0.058).

**Fig. 2:**
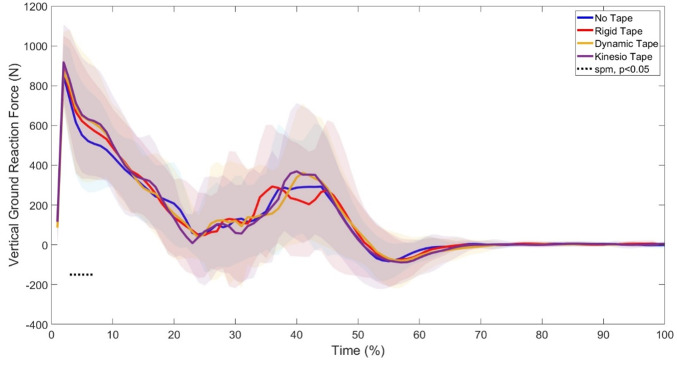
Tape (RT), Dynamic Tape (DT), and Kinesio Tape (KT) Conditions in twenty-one female netball players. Age: 22.0±2.7 Years; Height: 1.68±0.08 cm; Mass: 63.3±10.6 kg. Highlighted areas indicate significant changes.

**Fig. 3:**
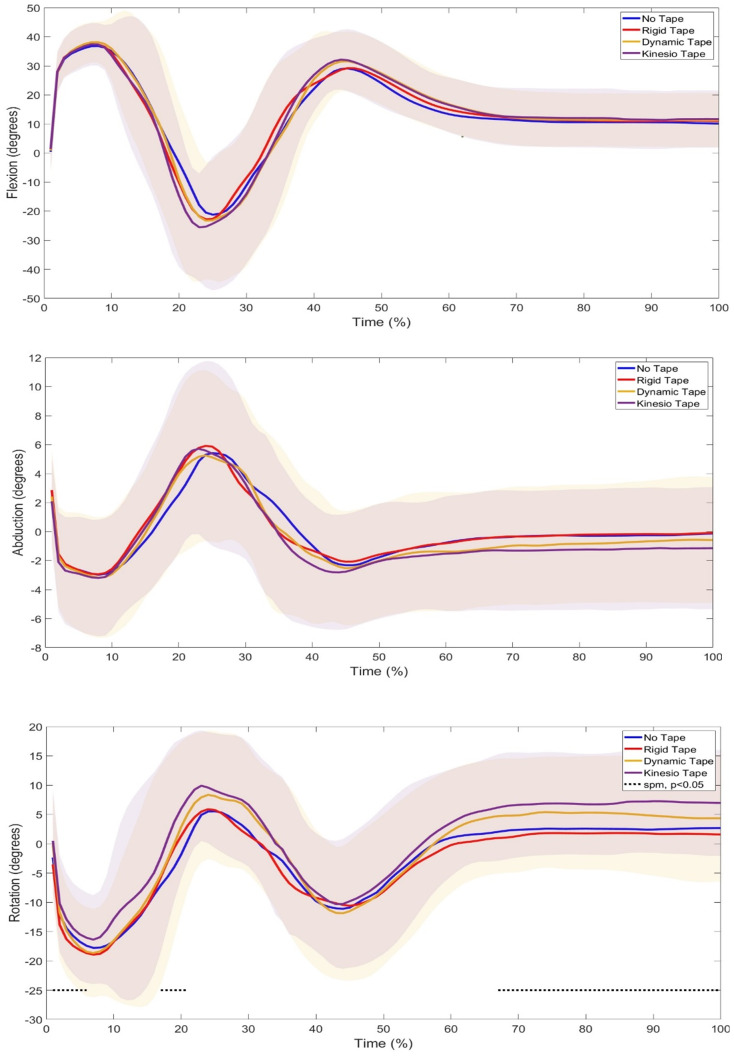
Comparison of Ankle Joint Angles during a 40cm Drop Jump Landing (DJL) across No Tape (NT), Rigid Tape (RT), Dynamic Tape (DT), and Kinesio Tape (KT) Conditions in twenty-one female netball players. Highlighted areas indicate significant changes.

**Fig. 4:**
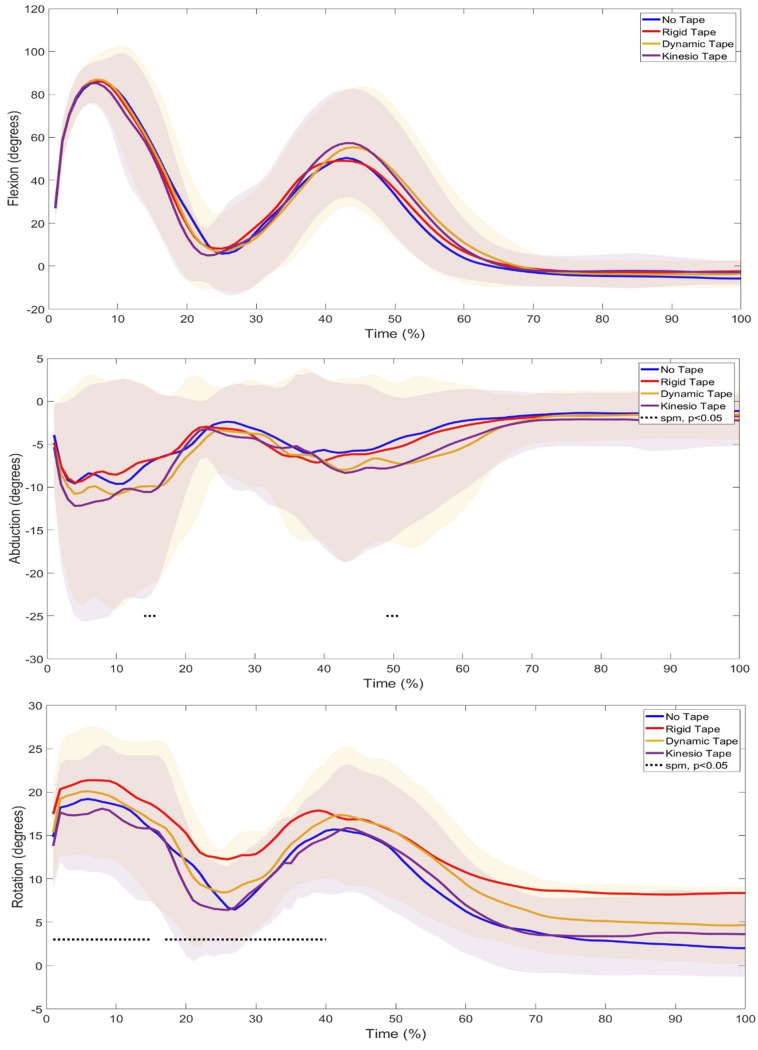
Comparison of Knee Joint Angles during a 40cm Drop Jump Landing (DJL) across No Tape (NT), Rigid Tape (RT), Dynamic Tape (DT), and Kinesio Tape (KT) Conditions in twenty-one female netball players. Highlighted areas indicate significant changes.

**Fig. 5:**
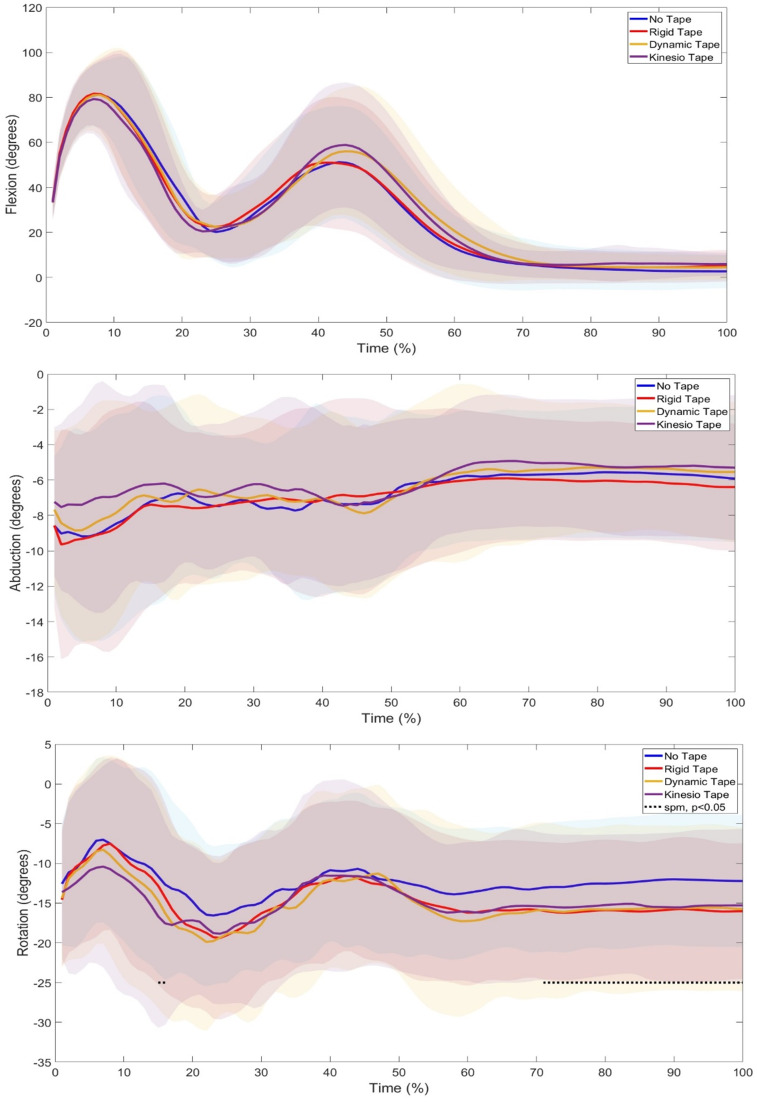
Comparison of Hip Joint Angles during a 40cm Drop Jump Landing (DJL) across No Tape (NT), Rigid Tape (RT), Dynamic Tape (DT), and Kinesio Tape (KT) Conditions in twenty-one female netball players. Highlighted areas indicate significant changes.

SPM1d analysis in the relative GRF showed a significant difference between 3–7% of the phase (p=0.001) (Figure 2) where Post Hoc tests revealed specific differences between NT and RT (p<0.0085) and NT and KT (p<0.0085). Further SPM1d analyses of joint kinematics and Post Hoc tests revealed significant changes in ankle rotation (phases 1–6%, p=0.01; 17–20%, p=0.02; 67–100%, p=0.01) (Figure 3) between NT vs KT (phase 17–20%), RT vs KT (phase 1–3%), and DT vs KT (phase 1–6%). Significant changes were also seen in knee abduction (phases 14–16%, p=0.02; 49–51%, p=0.03) between with NT vs DT (phase 14–15%) and NT vs KT (phases 49–52%; 54–64%) and rotation (phases 1–15%, p=0.02; 17–40%, p=0.01; 51–100%, p=0.01) between with NT vs RT (phases 1–19%; 22–39%; 49–100%), RT vs KT (phases 1%; 2–7%; 8–14%; 17–29%; 59–100%), RT vs DT (phase 91–100%), and DT vs KT (phase 1–10%; 37–41%) (Figure 4). Lastly, significant changes were seen in hip rotation (phases 16%, p=0.03; 71–100%, p=0.01) between NT vs RT (phases 64–67%; 69–100%) and NT vs KT (phase 84–100%) (Figure 5).

## Discussion

This study aimed to investigate the effects of different taping conditions on knee joint kinematics and reactive strength during a DJL. Significant changes were seen in GRF, GCT, and RSI. SPM1d analyses of joint kinematics revealed significant changes in ankle rotation, knee abduction and rotation, and hip rotation during the movement cycle.

GRF are strongly associated with injury risk, where an athlete’s ability to attenuate/absorb forces upon landing can translate into more efficient movement strategies, decreasing the risk of injury.^[12]^ Significant changes were seen in GRF in the eccentric and concentric phases of the jump after the first landing (Figure 2), where post-hoc tests further revealed significant differences between NT and RT, and NT and KT. When further analysing the GRF in the RT and KT conditions, it can be seen that the taping conditions did not influence force attenuation upon peak GRF on ground contact, but rather decreased the loading rate upon landing. Previous research using the Mulligans tape technique has shown that RT decreased GRF load upon landing, whereas KT had no effect,^[11]^ but the study used discrete analysis, only analysing the peak values, whereas the current study used discrete analysis and continuous analysis, analysing both the peak values and the change in GRFs through the entire movement cycle. The decrease in loading rate seen in the initial landing in the RT and KT conditions may indicate that the participants landed softer in comparison to the NT and DT conditions, as it has been found that soft landings induce an extended landing phase duration.^[11]^ An extended loading rate leads to more mechanical energy dissipation, decreasing stress on the joints and injury potential.^[11]^ While injury risk can be mitigated by taping,^[11]^ athletes must ensure that overall performance is not impeded, such as landing impact force, jump height, RSI, and GCT, which provide insight into an athlete’s explosive capabilities and efficiency of force transfer.

The landing impact force was revealed to be the highest in KT, followed by RT, DT, and NT (Table 1), revealing that the three taping conditions increased landing impact force compared to NT. The landing impact force represents the peak force exerted against the ground at the moment of take-off, after completing the eccentric phase and transitioning through the stretch-shortening cycle (SSC), where higher eccentric forces can enhance the SSC, leading to more elastic energy storage in tendons and muscles, and contribute to jump height.^[16]^ This relates to the jump heights (Table 1) as KT, RT, and DT increase jump height compared to NT. A high landing impact force indicates better power and reactive strength. An athlete’s ability to efficiently produce and transfer force during a jump is essential for performance, and RSI is a metric used to assess this.^[6]^

A higher RSI value correlates with better jump performance, characterised by increased jump height and shorter GCT. ^[5]^ Keeping this in mind, the RSI (Table 1) revealed significant changes between NT vs KT, where the KT condition increased RSI. Further, the DT and RT conditions, although not significant, also increased RSI compared to NT, suggesting that the three taping conditions increased RSI compared to NT. The RSI is considered to represent a fast SSC ability when GCT is less than 250 milliseconds,^[17]^ which was seen in all four conditions in this study. While the jump height was not significantly influenced by the taping conditions, there was a significant change in GCT (Table 1) where RT, DT, and KT reduced contact time compared to NT. It was further revealed that significant differences were seen in GCT between NT and KT. Although the change in RSI and GCT was statistically significant, the small effect size suggests that the magnitude of the change may have limited practical implications. However, in a sports and conditioning setting, even small changes can influence performance and injury risk. Overall, the reduction in GCT seen in the KT condition, contributed to KT being the highest in the RSI.

The kinetics observed reveal both injury prevention and performance enhancement implications. While the KT and RT conditions did not affect force attenuation at peak GRF, it lowered the loading rate, potentially increasing mechanical energy dissipation and reducing joint stress.^[12]^ It was also seen that the three taping conditions, compared to NT, improved performance metrics of landing impact force, jump height, GCT, and therefore RSI, with KT showing to have had the most performance improvement overall.

Kinematics are largely used to identify movement errors for injury prevention. Continuous kinematic data were analysed using SPM1d analyses. The results revealed significant changes in ankle rotation (Figure 3), where the Post Hoc tests revealed that the significant differences were in the eccentric and concentric phases between RT vs KT, DT vs KT, and NT vs KT. This suggests that KT caused the most ankle rotation. A similar pattern was observed in knee rotation (Figure 4) throughout the landing phases, which may have influenced the ankle, as it is seen that modification at one joint, such as the knee, may cause change to its neighbouring joint, such as the ankle.^[18]^ When analysing the normalised peak ankle angles (Table 2) from the eccentric to flight phase, significant differences, with a large effect size, were seen in ankle abduction, where it was further seen that there was a significant difference between DT and KT. Ankle abduction was decreased in the DT and KT conditions. Although not significant, it was seen that the knee taping conditions slightly increased ankle flexion and decreased ankle rotation. Previous research has shown the effects of ankle taping on ankle and knee joint angles,^[19]^ but there is a lack of research on the effects of knee taping on peak ankle joint angles. It can be said, however, that the knee taping condition’s effect on the peak ankle joint angles were not substantial.

The most change was seen at the knee joint, which was expected as this is where the taping conditions were applied. Landing from a jump is commonly used to evaluate the neuromuscular control of knee abduction.^[1]^ Significant changes were seen in knee abduction (Figure 4), where NT vs DT and NT vs KT caused more knee abduction in the concentric (first landing) and attenuation (second landing) phases of the DJL. DKV is an abnormal movement pattern visually characterised by excessive medial movement, such as adduction, of the lower extremity during tasks like landing.^[20]^ The increase in abduction seen in the DT and KT conditions may suggest a decrease in DKV upon landing. Previous studies have also found that DT and KT on the knee significantly decreased DKV^[10]^ and, although not significant, increased peak knee abduction angles during a DJL.^[21]^ Additionally, significant changes were seen in knee rotation (Figure 4) through all the jump phases, with significant differences seen between NT vs RT, RT vs KT, RT vs DT, and DT vs KT. This suggests that RT and DT kept the knee in a more rotated position throughout the phases. This can also be seen in the peak knee rotation angles (Table 2), where significant changes were found, with a large effect size, in RT vs KT and DT vs KT, where the RT and DT conditions increased knee rotation. The taping technique used for RT and DT was Mulligan’s taping technique, which has been shown to keep the knee in a rotated position upon application.^[22]^ This may explain the increased rotation seen in this joint. The peak knee flexion angles (Table 2), although not significant, showed that the RT, DT, and KT conditions all increased peak knee flexion angles compared to NT. This is similar to the finding by Collins, et. al.^[21]^ and Limroongreungrat and Boonkerd,^[8]^ which did not find significant differences in the KT versus NT conditions during a DJL, but the peak knee flexion angles did show an increase with the taped conditions. Increased knee flexion on landing has been shown to improve joint energy absorption, leading to a decreased risk of injury.^[23]^ In an NT condition, Bates, et al.^[1]^ found increased knee abduction during the first landing and decreased knee flexion during the second landing of a DJL. This pattern can also be noted in the current study in all conditions. In addition, there was found to be increased hip adduction during the first landing and decreased hip flexion during the second landing in an NT condition,^[1]^ which is the pattern the current study found; however, it was observed that the taping conditions caused significant changes in hip rotation.

Significant differences were revealed in hip rotation (Figure 5) with NT vs RT (attenuation and control phases) and NT vs KT (control phases). This suggests that RT and KT cause the hip to be in a more rotated position during these phases. Mackay, et al.^[22]^ found that the Mulligans technique at the knee using RT increased hip rotation during running. This may have had the same effect on the RT condition during landing. As for the KT condition, a similar pattern can be observed in the ankle and knee joints, where the joints were in a more rotated position in the attenuation and control phases of the movement cycle. As mentioned above, the modification seen at one joint can affect or alter its neighbouring joints,^[18]^ where the modification of the knee joint may have affected the ankle and hip joints.

The overall kinematics indicate that while all three taping conditions caused minor adjustments to joint angles, no single condition outperformed the other in joint angle modifications or impeded performance.

In the continuous analysis of the taping conditions compared to NT, it can be observed that KT had the greatest influence on improving the performance metrics, including GRF, GCT, and RSI. KT’s main mechanism of action is neuromuscular stimulation, which enhances joint proprioception. Proprioception plays a role in optimising movements, joint force, and joint position and is therefore considered an important protective mechanism, essential for athletic performance and physical well-being. [10] This performance improvement may, in turn, contribute to the athlete’s ability to rapidly generate force with a shorter GCT, which is essential for fast reactive movements in various sports.

### Implications

Overall, the joint kinematics revealed that while the taping conditions influenced ankle, knee, and hip joint angles, the statistical differences were not substantial enough to show that one taping condition outperformed the other in altering joint biomechanics. However, it can be noted that through the movement cycle, the RT condition followed a similar pattern to the NT condition, and the KT and DT conditions followed similar movement patterns to each other.

In terms of joint kinetics, the KT, RT, and DT reduced GCT but the overall reactive strength performance was highest in KT, leading to the highest RSI. Given KT’s neuromuscular stimulation properties, its performance benefits in the DJL may stem from enhanced proprioception, optimising joint positioning sense to minimise energy loss in the eccentric phase and maximise force production in the concentric phase. These results highlight the potential of knee taping in sport which utilise a large jumping component or use taping as an intervention for performance and should form one part of a broader performance enhancement strategy for athletes.

### Limitations

While this research offers valuable insights into reactive strength performance during DJL, a few limitations can be considered. The DJL protocol may not best simulate the random jumps and landings experienced in sports, and future studies may consider simulating different jump conditions when assessing for reactive strength. Additionally, although the tape was placed on the participants by the same practitioner, small variations in tension or placement could impact performance outcomes, highlighting the potential for human error. These may be of consideration in future studies.

## Conclusion

The DJL test is a valuable tool used to analyse reactive strength and jump performance in an athlete, providing insights that can inform targeted training and conditioning programmes aimed at enhancing explosive strength and injury prevention. Continuous analysis of the full DJL cycle revealed subtle kinetic and kinematic differences between taping conditions.

## Figures and Tables

**Table 1:** Reactive Strength Indexes during a 40cm Drop Jump Landing: Comparison of No Tape (NT), Rigid Tape (RT), Dynamic Tape (DT), and Kinesio Tape (KT) conditions in twenty-one female netball players

	NT	RT	DT	KT

Jump Height (m)	0.27±0.03	0.28±0.04	0.28±0.03	0.28±0.03
Ground contact time (ms)*	119.89±27.02	115.39±26.28	113.55±34.27	106.48±31.71†
RSI*	2.51±0.61	2.63±0.72	2.78±1.06	3.01±0.94†
Landing impact force (N)	935.51±210.07	960.16±224.93	956.32±282.26	988.21±227.51
Relative Landing Impact Force (N/kg)	15.24±2.56	15.46±2.63	15.13±3.24	15.79±2.85

* Friedman’s test, p<0.05.

† significantly different from no tape.

**Table 2:** Normalised peak joint angles of the right lower limb from Start to Flight phase during a 40cm Drop Jump Landing: Comparison of Rigid Tape (RT), Dynamic Tape (DT), and Kinesio Tape (KT) Conditions Normalised to the No Tape (NT) Condition

	RT	DT	KT

Ankle flexion	2.33±7.98	0.65±3.12	1.11±3.04
Ankle abduction*	0.09±0.88	−1.09±4.20†	−0.64±4.02
Ankle rotation	−1.28±3.23	−2.75±4.31	−2.43±4.42
Knee flexion	1.20±3.20	1.49±5.69	2.52±5.54
Knee abduction	−0.06±1.20	0.55±3.36	0.65±2.48
Knee rotation*	−2.41±3.26 †	−1.71±5.19 †	0.98±3.87
Hip flexion	−0.15±5.06	1.35±6.44	4.27±7.29
Hip abduction	0.40±2.29	0.03±3.88	−0.78±2.73
Hip rotation	1.16±2.24	0.86±4.73	1.61±5.51

* Friedman’s test, p<0.05.

† significantly different from KT.
